# Time, space, and disorder in the expanding proteome universe

**DOI:** 10.1002/pmic.201600399

**Published:** 2017-03-20

**Authors:** David‐Paul Minde, A. Keith Dunker, Kathryn S. Lilley

**Affiliations:** ^1^ Cambridge Systems Biology Centre University of Cambridge Cambridge UK; ^2^ Cambridge Centre for Proteomics Department of Biochemistry University of Cambridge Cambridge UK; ^3^ Department of Biochemistry University of Cambridge Cambridge UK; ^4^ Center for Computational Biology and Bioinformatics Indiana University School of Medicine Indianapolis IN USA

**Keywords:** Alternative splicing, Conformation, Intrinsically disordered protein, Membrane proteins, Post‐translational modification

## Abstract

Proteins are highly dynamic entities. Their myriad functions require specific structures, but proteins’ dynamic nature ranges all the way from the local mobility of their amino acid constituents to mobility within and well beyond single cells. A truly comprehensive view of the dynamic structural proteome includes: (i) alternative sequences, (ii) alternative conformations, (iii) alternative interactions with a range of biomolecules, (iv) cellular localizations, (v) alternative behaviors in different cell types. While these aspects have traditionally been explored one protein at a time, we highlight recently emerging global approaches that accelerate comprehensive insights into these facets of the dynamic nature of protein structure. Computational tools that integrate and expand on multiple orthogonal data types promise to enable the transition from a disjointed list of static snapshots to a structurally explicit understanding of the dynamics of cellular mechanisms.

## 1 Introduction

**Figure 1:**
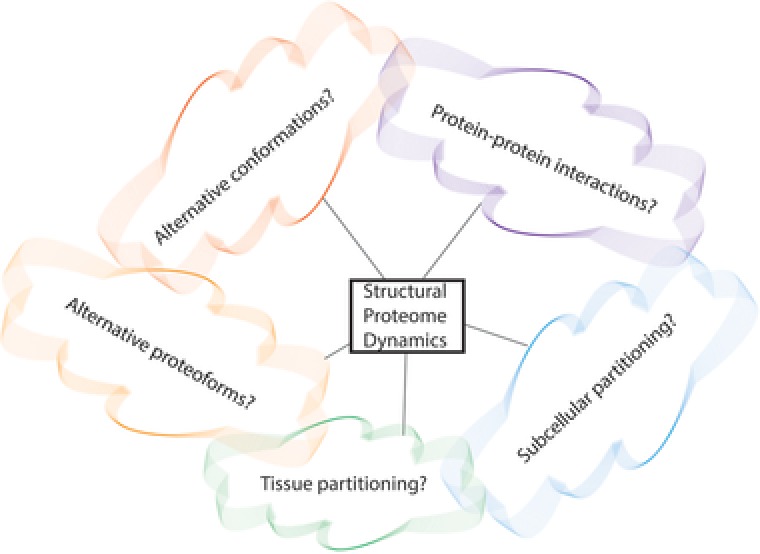
Challenging questions in proteomics. The proteome is not a fixed entity but a dynamic system. Unraveling a multitude of dynamic layers of its regulation is key to comprehensive understanding.

The human genome sequence has a smaller number of genes than expected: ∼19 000 compared to 6.7 million genes in earlier estimates 1. It has remained largely unclear how this small number of genes can be sufficient to support human complexity. In recent years, hierarchical layers of regulation have been revealed that give rise to some of the functional complexity observed in living cells despite the compact nature of the protein coding genome. These are directly linked to spatiotemporal dynamics on all levels of protein structure from their sequence, three‐dimensional structure to alternative cellular localizations and spatial organization of specific proteins in tissues and organs. We discuss these new regulatory mechanisms which contribute to emergent complexity of living systems (Fig. 1), as follows:

- About 80% of the human genome maps to non‐coding yet functional genomic elements 2. These regulatory elements include sites for DNA methylation, DNase I hypersensitive regions that function as preferential interaction sites for transcription factors and long‐range regulatory elements. Fine‐tuning the control of transcription makes it possible to switch among a large variety of transcriptional states depending on intracellular and extracellular changes.
- Alternative splicing has been implicated in tissue differentiation and is positively correlated with organism complexity 3, 4, 5. Alternative splicing is an important mechanism to generate multiple sequence variants from the same gene, for instance in different tissues or developmental stages 6.
- Post‐translational modifications (PTMs) such as phosphorylation 7 and acetylation 8 crucially modulate protein function. PTMs further expand the space of alternative sequence variants of proteins.
- Intrinsically disordered proteins (IDPs) and intrinsically disordered regions (IDRs) can assume alternative secondary and tertiary conformations. This expands the available space of alternative structures 9.
- Switchable alternative protein–protein interactions lead to yet more diversity 10. IDPs can engage in a large number of alternative interactions as function of PTM and overlapping short linear motifs. Some IDPs use overlapping linear segments for binding to multiple, distinct protein partners with low affinities 11, thus enabling rapid rewiring of large cellular interaction networks; these same capabilities enable the rapid rewiring of gene regulatory networks 6, 12, 13.
- Protein turnover. The half‐lives of eukaryotic proteins range from on the order of minutes to decades 14, 15. Such differential protein turnover leads to a greater range of protein abundance than for example, transcript abundance. Transcripts vary some 2 orders of magnitude in their cellular abundance whereas proteins cover a dynamic range greater than 6 orders of magnitude or higher in some cell types. Low‐abundant proteins are turned over more rapidly by proteasomal proteolysis, contain more IDRs and are enriched in PEST motifs 16, 17.
- Multiple subcellular locations enable the same protein to exert different functions in different parts of the cell 18.
- Proteins involved in transducing intrinsic and extrinsic signaling exist within spatially restricted concentration gradients. For example, Wnt signaling gradients control asymmetric cell divisions during early development and later in the life of complex organisms maintain tissue organization. Spatial organization enables the formation of complex tissues and organs up to the highly interconnected human brain.

Correspondence concerning this and other Viewpoint articles can be accessed on the journals' home page at: http://viewpoint.proteomics‐journal.de

Correspondence for posting on these pages is welcome and can also be submitted at this site.

The term “proteoform” has been recently proposed as an umbrella term to summarize all possible alternative protein sequences for a given protein including genetic sequence variants, PTMs, splice variants, proteolysis variants 19. Powerful methods to characterize proteoforms have been comprehensively covered in several excellent papers 20, 21, 22. It should be added at this point that IDPs and IDRs can be viewed as “outliers” in the context of structural biology terminology: Folded proteins are readily described by the well‐established hierarchy of 1D structure (i.e. protein sequence) to 2D structure (i.e. local secondary structure elements) to 3D structure (i.e. atomic coordinates of atoms of a folded protein chain), but IDPs lack a fixed 2D or 3D structure and therefore elude a straightforward classification in the established terminology framework. To cope with this phenomenon, it was recently suggested to extend the concept of “proteoforms” to include manifold alternative conformations of IDPs and IDRs as “conformational (or basic or intrinsic) proteoforms” 23. Other authors have used various descriptors for IDPs, 24 including “4D proteins” 25 to indicate that their conformations and functions can change over time or, alternatively, other authors have attempted to classify IDPs by physical parameters such as charge patterns, IDR length and residual structure 26. While an in‐depth discussion of the issue of IDP classification and terminology is clearly beyond the scope of this viewpoint, it is important to acknowledge the current imperfections of our terminology and to encourage community‐wide efforts to find a new consensus solution for a more effective terminology that would fully integrate IDPs and IDRs into the terminology of biological sciences.

Compared to extensive insights into multiple aspects of proteoforms, much less is known about higher‐order structural proteome dynamics that enable cellular complexity (Fig. 1). We focus on recently developed methodologies designed to study dynamic protein conformations, interactions, and subcellular mobility. We also present a brief summary of what we consider to be remaining key challenges in studying the structure and function of cellular proteomes.

## 2 How can alternative structures tune functional protein interactions (and *vice versa*)?

Not all protein interactions fit the classical lock and key model of molecular recognition achieved by docking of rigid components. Fine‐tuning target recognition can require ‘conformability’ as in the case of bacterial Lac repressor protein, which assumes a fuzzy complex when sliding along non‐specific DNA sequence but a mostly structured state in the specific, tightly DNA‐bound complex once associated with its specific target sequence 27. Similar observations have been made for human sequence‐specific transcription factor LEF1 which is mostly disordered free in solution but assumes a defined 3D structure in complex with its specific target DNA 28. Even more pronounced structural transitions from unstructured to pathologically structured fibril conformations can contribute to neurodegenerative disorders as in the case of Parkinson disease, which is associated with toxic accumulation of α‐Synuclein aggregates 29. Many cell‐regulatory hub proteins contain IDRs 30. Adenomatosis polyposis coli (APC), a tumor suppressor protein, is frequently mutated in cancer, and cancer mutated forms of APC often lack most of their 2000 residue long IDR. Axin1, an interaction partner of APC, can gain pathological functions if single point mutations disrupt the normal fold of a small folded domain that is located between its long IDRs 31, 32, 33. Increasing largely anecdotal evidence suggests that both transient and persistent structural disorder play crucial roles in biology and understanding disease mechanisms and that there is no unique disordered state but rather a continuum from fully structured to fully disordered 34, 35.

## 3 “LEGO brick” structural biology is getting more dynamic

### 3.1 X‐ray crystallography beyond static structures

Traditionally, structural biology was rationalized by the dogma that biological function requires a rigid 3D protein structure. According to this dogma, it should be possible to understand biology by solving one minimal energy structure per protein. Greater than 100 000 structures of folded domains have been solved over the last decades and first near‐complete structural proteome models have been proposed based on homology modeling 36. 90% of these protein structures have been solved using X‐ray crystallography, which is intrinsically restricted to the solid phase of proteins. Structural protein dynamics in solution are, therefore, incompletely characterized so far.

Despite its historical bias towards solving static structures, X‐ray crystallography has chiefly contributed to the birth of the IDP field 37 as thousands of polypeptide segments in crystallised protein constructs do not give rise to a well‐defined electron density and can therefore be classified as “disordered” 38. More direct time‐resolved methods are currently under development building on the latest advances in high‐brilliance X‐ray sources. Spectacular first dynamic pictures of ultrafast light‐induced femtosecond isomerization events in the photoactive yellow protein and alternative conformations of riboswitches dynamically reshaping upon ligand‐binding highlight the possibility of capturing dynamic structural data in the future 39, 40. In addition to exciting technological developments, it will be interesting to explore improved computational possibilities for a more comprehensive analysis of existing X‐ray crystallographic datasets: further improvements are possible by treating protein dynamics explicitly and enabling improved fitting of existing electron density maps to alternative conformations and locally flexible parts in proteins 41.

Even fully disordered proteins are no longer outside of the reach of X‐ray crystallography. Several important c‐Myc structures have been solved in complex with specifically binding partner proteins 42. It is hoped that this will make previously undruggable IDPs specifically targetable by exploiting unique interfaces that only arise in specific protein–protein complexes of these IDPs 43. X‐rays can make numerous protein dynamics crystal clear.

### 3.2 Cryo‐EM and NMR – a dynamic pair

In the last two decades, major technological breakthroughs in electron detection efficiency and image processing have culminated in a recent explosion of new Cryo‐EM structures, which is experiencing a higher average annual growth compared to x‐ray crystallography (with an average of 34 versus 9%). Cryo‐EM, like NMR spectroscopy, is capable of revealing local structural disorder. NMR peaks of disordered protein segments cluster together more closely because their more averaged chemical environments result in lower chemical shift dispersion 44. Anisotropic Cryo‐EM resolution scales with flexibility 45, i.e. highest resolution is achievable for rigid and lowest resolution for very flexible regions 46. Their preferred molecular size ranges are complementary: typically below 50 kDa for NMR and above 150 kDa for Cryo‐EM. While the rate of progress is nicely accelerating, costs of state of the art Cryo‐EM and NMR facilities still restrict broader community access to these technologies. Establishing optimal protein production protocols and sample conditions remain shared bottle‐necks among all high‐resolution structural techniques 47, 48. While the contribution of NMR to solving new structures might shrink in the future, it cannot be over‐emphasised that this technique has unique capabilities in covering directly a large range of protein solution dynamics on timescales ranging from picoseconds to hours 49. Briefly, all major high‐resolution structural biology technologies continue to develop dynamically and complement each other.

### 3.3 Biochemical approaches to study protein conformational dynamics

Many aspects of protein conformational dynamics are either impractical or impossible to study using exclusively above‐mentioned high‐resolution structural methods. Biochemical methods including 1D SDS‐PAGE and proteolysis have been successfully used as valuable complementary methods to characterize protein folding and conformational heterogeneity in solution 50. Short digestion protocols as in pulse proteolysis 51, 52, membrane pulse proteolysis 53, SILAC pulse proteolysis 54, and FASTpp 55 considerably increased throughput in recent years. FASTpp uses thermal denaturation in contrast to chemical denaturation in pulse proteolysis. FASTpp exploits the principle of rapid digestion of exposed, thermally unfolded polypeptide segments before they had a chance to aggregate. FASTpp detects ligand‐induced folding and stabilisation, missense mutation effects on protein stability 56, 57, 58. While FASTpp is technically simple and fast to implement without the need to equilibrate samples in denaturant, which can take months in the case of kinetically stable proteins 59, pulse proteolysis can be used to derive equilibrium unfolding energies (ΔΔGs).

Limited proteolysis (LiP) has been used for many decades in structural biology and continues to be actively developed using a wide range of proteases and readout methods from low to high multiplexity 60, 61. A recent breakthrough study used LiP in combination with peptide sequencing by mass spectrometry to simultaneously map conformations of 1000 yeast proteins and to reveal quantitative structural changes in 300 proteins upon growth on different sugars 62, 63. Similar methods combining the best of classical biochemical methods and ultra‐sensitive and large‐scale protein detection have a great potential for revealing structural proteome dynamics under a large range of biological 64, physical, and chemical conditions, thereby redefining our understanding of protein stability and folding in the cellular context.

### 3.4 Label‐dependent protein folding assays

A wide range of highly specific methods to study protein conformations depends on selective chemical protein labeling. Tryptophan‐free proteins can be selectively labeled using a single tryptophan substitution of a chemically similar aromatic residue like phenylalanine, which often does not perturb the biological behavior of the wild‐type protein 65, 66. Another more widely used chemical labeling method is hydrogen deuterium exchange (HDX). As all proteins contain hydrogens, their exchange with deuterons presents a very generic and minimally perturbing strategy of labeling. Hydrogens are ubiquitous in proteins yet local hydrogen to deuterium exchange rates vary over many orders of magnitude depending on their structural interactions: rigidly folded and hydrogen‐bonded segments of proteins exchange very slowly (∼hours to years) while random coil regions can often exchange rapidly (∼milliseconds–seconds) 67. This effect can be used to investigate how much structure a disordered region assumes upon addition of specific ligands by investigating how the exchange rates decrease as ligand is added. A recent study demonstrated the use of reverse (i.e. deuterium to hydrogen) exchange to map peptidome‐wide peptide–protein interactions. This study highlights the fundamental possibility of exploiting atomic changes to map protein interactions on a global scale 68. Using HDX technologies on whole cells for cellular structural studies is a desirable extension of the method, however a significant hurdle is the need to minimize back‐exchange during necessary processing steps such as cell lysis and protein digestion prior to bottom‐up LC‐MS/MS analysis. Novel strategies in directed evolution or metagenomics selection 69 have the potential to identify novel types of acid‐compatible specific proteases that can help to accelerate specific digestion under conditions that drastically slow down back‐exchange. These highly acidic conditions would be only necessary after conformational features are “encoded” as deuterium incorporation and thus do not affect the native structural states of cells. Ultra‐rapid digestion methods, mass‐spectrometry compatible detergents and faster computation of complex spectra resulting from a large number of variable isotope changes may further help to pave the way toward proteome‐wide in vivo HDX experiments 70, 71.

### 3.5 Solubility methods to probe protein conformation

Alternative methods based on physical principles increasingly complement chemical methods. One of the earliest physical methods to characterize protein unfolding is monitoring their soluble fraction at a range of temperatures. Analogous to egg‐white protein in boiled eggs, most proteins irreversibly precipitate above their unfolding temperature. Temperatures just slightly above the physiological growth optimum can cause dramatic reductions of proteome solubility in cells lacking the Hsp70 system that is an essential component of the cellular heat shock protection system by interacting with aggregation‐prone unfolded and partially folded proteins 72, 73. The cellular thermal shift assay (CETSA) assay exploits this effect to screen ligand‐dependent changes of thermal solubility of proteins 74. CETSA revealed drug‐dependent increases of kinase stability. Initial examples of CETSA required a large number of samples to be screened by quantitative antibody‐based detection methods 74. Thermal proteome profiling (TPP) overcomes the dependence on antibodies and limited throughput by combining the CETSA principle with TMT 10‐plex mass spectrometric detection in a large temperature window between 37 and 67°C. A small number of TPP runs in human cells and cell lysates enabled quantitatively tracing drug interactions with nearly 7000 human proteins and revealed off‐target interactions of a drug 75.

Interestingly, TPP can be also applied to many transmembrane proteins either before or after detergent solubilisation using a range of mild detergents 76. A systematic comparison of both datasets suggests that cellular compartments alter the biophysical stability of membrane proteins: membrane proteins in native membranes are more stable than intracellular proteins while detergent‐solubilized membrane‐proteins are less stable compared to intracellular proteins. This finding suggests that membrane proteins are more stable in vivo than intracellular proteins yet significantly less stable in vitro consistent with their reputation of being notoriously unstable during crystallization trials in detergents. As protein structural dynamics affect protein interactions and interactions in turn affect structural stability, characterizing these dynamics‐functional relations is of fundamental interest and has started to be bio‐medically transformative by establishing novel drug discovery routes.

### 3.6 How can transient protein–protein interactions contribute to functional diversity?

Specific protein‐protein interactions (PPI) are widely considered as key to understanding cellular functions of proteins. One might intuitively expect most PPI to be high affinity as this ensures a high fraction of specifically bound complexes. Highest affinity can be reached with rigid proteins, but transient and biophysically weak interactions are at the hub of biological interaction networks 77 and ultra‐affinity is rare 78. One of the most striking examples for two non‐rigid proteins interacting specifically is the mutually synergistic folding of two independently flexible proteins in the ACTR‐NCBD complex 79. Both protein domains engage in an intimate complex that covers a large, rather hydrophobic interface to jointly regulate transcription as crucial parts of a large number of larger proteinaceous transcription‐regulatory machineries 80, 81.

### 3.7 High‐throughput affinity‐based methods to study protein interactions

Most large‐scale methods depend on short, disordered affinity‐tags 48, 82. Affinity purification (AP)‐MS uses a single affinity enrichment step and investigates all co‐eluting proteins, while tandem (T)AP‐MS uses two sequential affinity steps. More specific interactors than in sequential multiple‐affinity methods can be retrieved using two or more orthogonal tag systems in parallel for the same target protein, for instance FLAG‐tag and Strep‐tag, in interactomes using parallel affinity capture (iPAC) 83 or quantitative SILAC‐iPAC 84. Recently, GFP was introduced as novel affinity tag in AP‐MS 85, which made it possible to build on existing large GFP‐fusion libraries and to selectively enrich interactors of most human proteins 77.

How good are these methods for capturing weak yet potentially biologically important interactions? It is a priori not clear how these methods might bias against the detection of very transient binding events shorter than current affinity protocols or bias toward complexes that only form in vitro in dilute lysis and affinity purification buffers but would never form in the crowded intracellular environment in the presence of optimal concentrations of molecular chaperones. Clearly, orthogonal methods are needed to validate interactions and to discover additional interactions that are too transient or weak for detection by affinity‐enrichment methods.

### 3.8 Overcoming the quantitative protein‐protein interaction validation bottle‐neck

While affinity methods readily provide large lists of specific interactors, it is generally difficult to derive predictions about proteoform‐specific dissociation constants, which would enable quantitative predictions for other protein concentrations. Direct biophysical high‐throughput quantification of binding strength of putative protein‐protein interactions has remained highly challenging. Single‐molecular‐interaction sequencing (SMI‐seq) enables high‐throughput quantification of up to hundreds of protein interactions in parallel covering a broad range of affinities by covalently crosslinking proteins to nucleotide‐barcodes for multiplexed sequencing in situ 86. SMI‐seq has been successfully applied to both water‐soluble and membrane proteins incorporated in phospholipid bilayer nanodiscs 86. SMI‐seq uses cell‐free in vitro production of proteins and is, therefore, not fundamentally limited by the natural genetic code. Related approaches that offer high‐throughput and quantification of protein interactions will be valuable for coping with the validation bottle‐neck in protein‐protein interaction research.

### 3.9 Comparing in vitro and in vivo protein associations

Comparison of in vitro and in vivo protein complexes is in principle possible by fixation of protein interactions using chemical crosslinking or using fluorescence correlation spectroscopy (FCS) 87. In vivo FCS can visualize the dynamic assembly and disassembly of protein complexes during the cell cycle 87. Crosslinking mass spectrometry (XL‐MS) recently advanced from the study of a few crosslinks of small protein complexes to large viruses thanks to improvements on all levels from MS‐cleavable cross‐linkers over new mass spectrometric strategies to novel data analysis workflows 88, 89, 90. A wide range of cross‐linkers exist that cover zero‐length to several nanometers in distance between crosslinked molecules. Relatively short lengths, such as 0.5 nm for MS‐cleavable DSSO, can be ideal for use in integrative biology to refine structural models of protein complexes of partly solved composition 91. Larger cross‐linkers can be beneficial to elucidate the network of transiently or weakly binding proteins in large protein complexes 92. Future expansion of these novel crosslinking‐MS strategies to in vivo analysis of intracellular protein complexes using a class of cross‐linkers that combines clickable affinity purification handles for enrichment of crosslinked peptides and MS‐cleavability for accelerated peptide identification is becoming possible 93, 94.

### 3.10 How does protein‐organelle partitioning affect protein interactions?

Even the simplest known living cells are compartmentalized 95. Membrane enrichment is crucial for membrane‐intrinsic transporters and helps to orchestrate a variety of metabolic pathways 96. Eukaryotic cells have multiple membrane‐enclosed organelles that enable a wide range of physicochemical conditions to coexist in a single cell. Secretory granules can have a pH of 5.0 while other compartments typically vary between pH 6.4 and pH 7.2 97. Some proteins are fully folded in one compartment but unfolded in another 98.

### 3.11 Chemical proximity‐labeling strategies to discover protein co‐localisation

Efficient strategies are being developed to selectively label membrane‐associated protein complexes for subsequent MS detection. APEX2‐MS 99 is based on an enzyme that catalyzes the conversion of biotin phenol to a biotin radical and rapid labeling of nearby proteins 99, 100. Both phenol and peroxide as co‐substrates of this labeling reaction might induce cellular stress in some organisms and cell types. Selective proteomic proximity labeling using tyramide (SPPLAT) is a chemical variation to the same theme of enzymatically creating an activated biotin‐conjugate that has a short half‐life and therefore can only react in the immediate vicinity of the activating enzyme 101, 102, which is horse‐radish peroxidase in the case of SPPLAT in contrast to ascorbate peroxidase in APEX 103.

Biotinylation is in principle also possible using more gentle enzymatic approaches as biotinylation is one of the most specific known PTMs 104. This natural specificity is, however, a challenge for APEX‐like applications that require promiscuous biotinylation in the proximity of the enzyme. A mutant of the bacterial BirA ligase that lacks this substrate specificity, “BioID”, has been applied to discover transient interaction partners of specific BirA‐mutant labelled proteins 105; an accelerated unspecific biotin‐ligase called BioID2 is available 106, 107. Directed evolution might further improve the activity of BioID2 at 37°C as BioID2 is derived from a highly thermophilic (*Aquifex aelicus*) source and displays optimal activity far above 37°C 106. Additional improvements of the method appear possibly for many applications if biotin‐enrichment is performed on the peptide level instead of protein level as ∼200‐fold increased direct mass spectrometric detection was demonstrated for biotin‐peptides 108.

### 3.12 How does lipid‐less subcellular partitioning affect protein interactions?

Even within a single organelle, biomolecules are not homogenously mixed. Active sub‐organellar partitioning often involves ATP‐fuelled molecular machines, for instance dynein guiding cargo proteins along the cytoskeleton 109. Other sub‐organellar structures form spontaneously. IDPs have been recently identified as crucial components driving the assembly of membrane‐less cellular compartments. The prion‐like domain of Xvelo, an IDP, is crucial for formation Balbiani bodies that are a hallmark of asymmetry in oocyte formation 110. A variety of different flavours of protein‐RNA bodies have been identified including stress granules, nucleoli, Cajal bodies, and PML bodies in the nucleus. Intriguingly, some of their properties can be explained by sequence patterns in their specific IDPs. Specific F/R/G‐rich motifs in these IDPs can efficiently drive liquid‐liquid phase separations and contribute to formation of these membrane‐less bodies 111. Thus subcellular order comes, at least in part, out of intrinsic disorder.

Given their large molecular size, the ribosome and other large cellular machines including the proteasome and chaperonins constitute nanoscopic cellular compartments in their own right. Based on RNA‐seq and isolation of translationally halted ribosomes, and one‐by‐one addition of chaperones, it is now becoming possible to selectively profile ribosomal complexes to unravel how molecular chaperones engage during the translation process. This “selective ribosome profiling” approach revealed that trigger factor (TF) engages in vivo only upon emergence of ∼100 nascent residues in contrast to the earlier suggestions based on in vitro work on TF that TF is waiting per default at the ribosomal exit tunnel 112; analogous approaches have great potential to transform our understanding of spatiotemporal organisation of proteostasis including synthesis and folding of membrane proteins.

Exciting open questions related to suborganellar cellular structures include: how is the timing of metabolic pathways tuned by subcellular structures? Are PTMs regulating their formation? How can we monitor systems‐wide perturbations of these structures by changing environments?

### 3.13 Organelle proteomics

**Figure 2:**
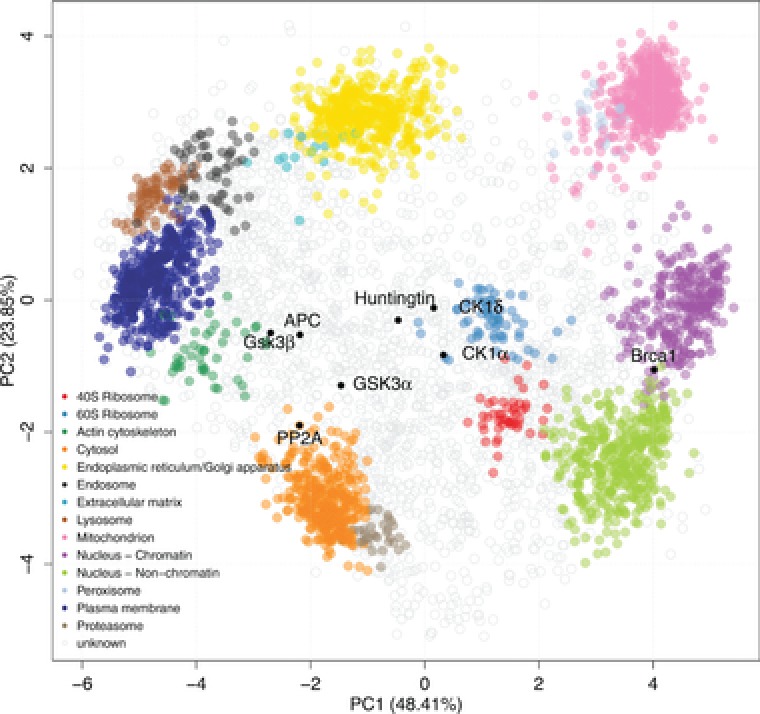
Thousands of proteins have multiple alternative cellular localizations 114, 118. Mouse stem cell hyperLOPIT data 114. Predominant variations of the partitioning of individual proteins into fractions of the density gradient are captured by the first two components (denoted PC1 and PC2) of a principal component analysis (PCA). Wnt signaling proteins APC2, CK1, GSK3β, neurodegeneration‐linked Huntingtin, and the breast cancer‐linked tumor suppressor protein BRCA1 (highlighted as solid black circles) are not assigned to a single location, characteristic of proteins with mixed localization.

Combining state of the art mass spectrometry, partial separation of organelles in a density gradient, and statistical analysis of resulting patterns enabled first quantitative and nearly proteome‐wide maps of cellular localizations for eukaryotic cells, such methods include protein correlation profiling (PCP) 113 and localization of organelle proteins by isotope tagging (LOPIT) 18. LOPIT has been further refined by combination with 10‐plex TMT labeling in hyper‐LOPIT 114. TMT labeling of peptides is independent of subcellular protein fractionation in density gradients and solely used to achieve maximal subcellular resolution, coverage of sub‐cellular niches and reduction of false assignments to different sub‐cellular niches; differential centrifugation and in‐solution digests have been used as technical variations of hyperLOPIT 115. LOPIT studies have revealed that many more proteins than expected are present in multiple locations of the cell. This observation gives rise to intriguing questions including how multiple locations are linked to structural and functional diversity and PTMs as well as splice variants and IDRs. APC, which contains an unstructured region of some 2000 residues, for instance, can travel from the nucleus to near the membrane and engage in several condition‐dependent transient functional protein and protein‐RNA complexes including the machinery for its own synthesis 116, 117. It will be a fascinating challenge to explore globally how other IDPs act differently in different parts of the cell and how dynamic cellular structure form under direct control from IDP regions. Selected examples for other Wnt pathway members are highlighted in a HyperLOPIT plot (Fig. 2) 118.

Despite their current limitations to relatively small numbers of different proteins that can be observed simultaneously, it will be interesting to explore the complementary benefits of cryo‐electron tomography (cryo‐ET) 119, 120, 121 and super‐resolution (SR) fluorescence microscopy 122. Both techniques are experiencing rapid technological advances and further improvements have the potential to provide novel insights into high‐resolution spatiotemporal subcellular dynamics as well as fine details of tissue architectures 123.

### 3.14 How can tissue and organ partitioning affect localized interactions?

Complex tissues and organs such as the human brain clearly require a high degree of spatial organization beyond single cells. Nearly 50 years ago, Francis Crick proposed diffusive “morphogen” gradients as minimal ingredient for spatial organization of cells during embryogenesis 124. Only very recently, it has become possible to directly visualize morphogen gradients in vivo using elegant organoid models that reflect most architectural features of organs while adding benefits of infinite expansion and culturability. Surprisingly, the measured short‐range cellular Wnt gradients are inconsistent with free diffusion but appear to require a cell‐bound propagation mechanism 125.

Wnt signaling as a whole is a perfect illustration of the importance of various levels of disorder in establishing multi‐cellular order. Many of its crucial signaling components including the scaffolds APC, Axin and WTX contain large IDRs up to some 2000 residues 33, have large numbers of PTMs and alternative interactions 126, are cellularly mobile (Fig. 2) and read the gradient signal that spans across several cell length and ultimately established tissue and organ shape. Curiously, the massively disordered APC protein is also needed for proper synapse formation in the brain. Specific mutations of APC correlate with autism and a conditional knock‐out impaired synapse maturation 127. Defined disorder appears to be an architectural hallmark of some of the most intricate structures in nature, which are just becoming observable by mass spectrometry imaging 128.

### 3.15 Computational biology helping to fill the voids in structural proteomics

Acquiring all‐atom movies of the living organisms is clearly beyond experimental reach. Computational methods increasingly help to fill gaps in our understanding of structural biology. Efficient algorithms can predict secondary structure, IDRs and increasingly 3D structure can be predicted from readily available genomic sequences 129, 130, 131. Despite the astronomic conformational possibilities to arrange a given short polypeptide sequence in 3D, de novo prediction of the folding of ∼100 residue long peptides based on physical principles in silico has been shown for some examples 132. However, the community experiment on protein structure prediction known as CASP shows that de novo prediction of even small, single domain proteins, while improving over time, is still far from routine, and further shows that the most reliable method for protein 3D structure prediction remains the construction of protein models using the known structures of homologous proteins as templates. These template‐based models suffer from template bias, e.g. the resulting structures are more similar to the templates than to the true structures. Improvements in protein dynamics methods are finally leading to approaches for reducing the degree of template bias 133. Similarly, the most recent force‐field developments now show promise toward correct prediction of conformational ensemble properties of IDPs 134, 135.

Computational approaches can amplify the attainable insight from highly complex multi‐dimensional proteomics experiments by efficient dimensionality reduction methods. PCA plots often capture most of the variation of highly dimensional data in visually intuitive two‐dimensional plots (Fig. 2) 136. Significant computational science community efforts are needed to maximize the knowledge gain from rapidly accumulating and diversifying multi‐omics datasets to ultimately reveal fascinating new hidden ordered patterns in complex cellular dynamic systems 137.

### 3.16 Outstanding challenges in proteomics

 

- Which weak or transient interactions are functionally important?
- How to quantitatively understand and predict in vivo versus in vitro protein interactions?
- How can we quantitatively link various “omics” from DNA to RNA and the higher‐order structure of proteins including their cellular trafficking?
- What are the underlying principles determining cellular protein structural dynamics and how to predict them from readily accessible genomic sequences?
- How can we improve the mutually enhancing efforts of experimentalists and theoretical scientists to tackle highly complex “multi‐omics” projects? It is a formidable challenge for computational biologists and mathematicians to glean sufficient breadth of data types from experimentalists, to discover overarching patterns in various “omics” datasets that are fundamentally connected by common cellular biology.
- How can we link different protein structural states to functional diversity? The decade‐old C‐value paradox states that genome sizes are not well‐correlated with organism complexity 138. Extensive multi‐purposing in eukaryotic proteomes might explain the exceptional “coding efficiency” in many eukaryotic genomes that are too small relative to their complexity 4, 13. Quantifying the extent of multi‐purposing is highly challenging as individual dimensions such as PTM, alternative splicing and IDR discovery, and protein function prediction and validation are individually challenging. Expanding and integrating these efforts into comprehensive high‐throughput methods is highly desirable but not yet straightforward 139.
- Can we use our improved understanding of spatiotemporal proteome dynamics to improve life of ageing and growing societies?

## 4 Conclusion

**Figure 3:**
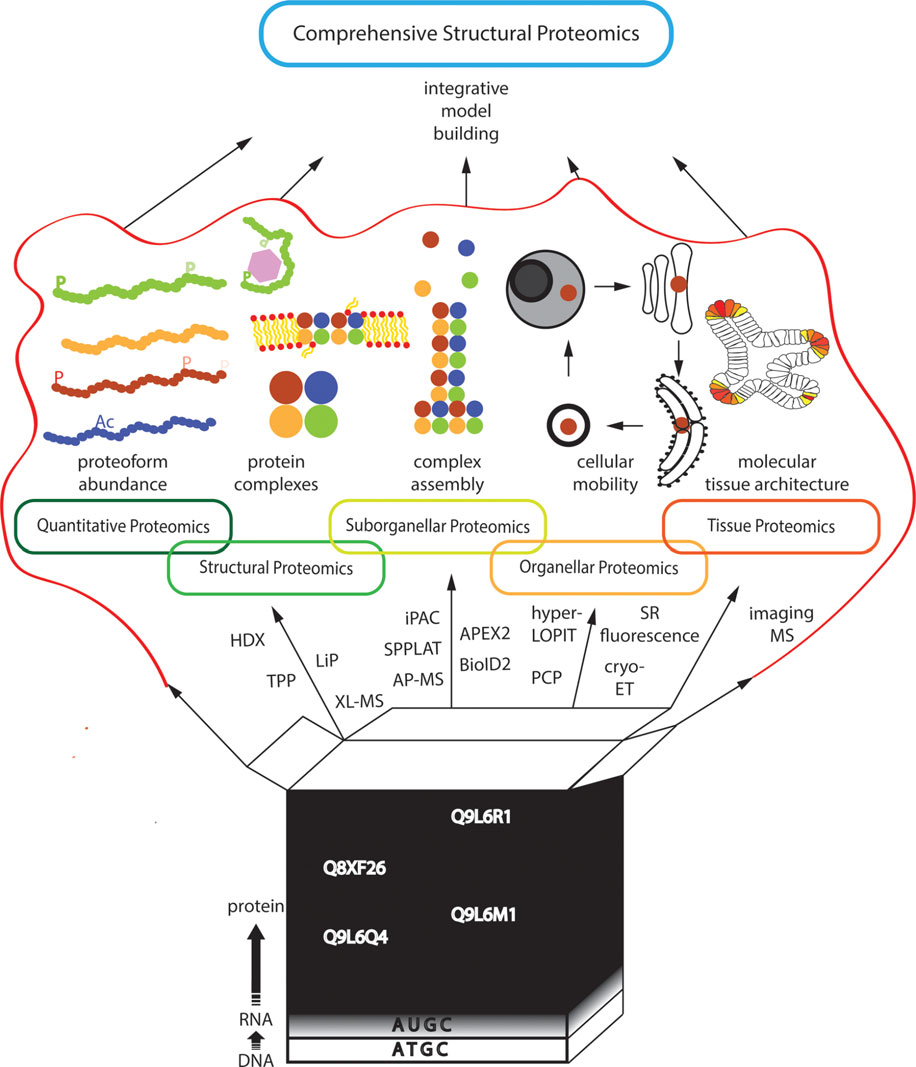
Opening the black box of biology. New proteomics techniques enable a more comprehensive understanding of the systems dynamics of life. New structural proteomics technologies can accelerate the analysis of the dynamics of biological regulation and extend the scope of structural biology well beyond its descriptive origins toward closer connections with cellular functions and prediction of systems behaviors of proteins.

Bottom‐up approaches have been very powerful in structural biology over many decades. DNA and RNA sequencing technologies have become highly robust and widely accessible technologies and rapid proteome‐wide protein sequencing is now possible for several organisms and transform our understanding of biology. Protein de novo folding simulations have reached near‐atomic precision for small folded domains and IDRs. Higher‐order structures are less readily predictable so far. Complicating factors are the intracellular and environmental fluctuations, which can be observed even in the most simple model systems 140, 141. Clever combinations of traditional biochemical and physical assays with increasingly rapid bottom‐up mass spectrometry generate many new opportunities to characterize these higher‐order structures as outlined in this review (Fig. 3). Collectively, these new bottom‐up mass spectrometric techniques make it possible to “sequence” many crucial layers of dynamic regulation of protein structures.

Very recent breakthrough studies demonstrated the possibility of few‐protein spatiotemporal engineering of organisms to improve carbon fixation or accelerate the process of switching from reduced photosynthetic activity under low‐light conditions to full photosynthetic productivity once more light becomes available after clouds have passed 142, 143. An improved proteome‐wide understanding of the hidden order in apparent disorder of higher‐order protein structures in living organisms can pave the way to de novo spatiotemporal engineering of organisms with beneficial properties. While this might sound like a long way off at present, it was well beyond the wildest imaginations just 20 years ago that we would be able to routinely sequence entire proteomes in an hour of measurement time 144. It will become increasingly possible to avoid late‐stage failures in drug discovery pipelines due to an improved understanding of cellular dynamics.

Plenty of dynamics at the bottom of biology (Fig. 3).

## References

[1] Pertea, M. , Salzberg, S. L. , Between a chicken and a grape: estimating the number of human genes. Genome Biol. 2010, 11, 206. 20441615 10.1186/gb-2010-11-5-206 PMC2898077

[2] The ENCODE Project Consortium , An integrated encyclopedia of DNA elements in the human genome. Nature 2012, 489, 57–74. 22955616 10.1038/nature11247 PMC3439153

[3] Xu, Q. , Modrek, B. , Lee, C. , Genome‐wide detection of tissue‐specific alternative splicing in the human transcriptome. Nucleic Acids Res. 2002, 30, 3754–3766. 12202761 10.1093/nar/gkf492 PMC137414

[4] Niklas, K. J. , Bondos, S. E. , Dunker, A. K. , Newman, S. A. , Rethinking gene regulatory networks in light of alternative splicing, intrinsically disordered protein domains, and post‐translational modifications. Front Cell Dev. Biol. 2015, 3, 8. 25767796 10.3389/fcell.2015.00008 PMC4341551

[5] Chen, L. , Bush, S. J. , Tovar‐Corona, J. M. , Castillo‐Morales, A. , Urrutia, A. O. , Correcting for differential transcript coverage reveals a strong relationship between alternative splicing and organism complexity. Mol. Biol. Evol. 2014, 31, 1402–1413. 24682283 10.1093/molbev/msu083 PMC4032128

[6] Buljan, M. , Chalancon, G. , Dunker, A. K. , Bateman, A. et al., Alternative splicing of intrinsically disordered regions and rewiring of protein interactions. Curr. Opin Struct. Biol. 2013, 23, 443–450. 23706950 10.1016/j.sbi.2013.03.006

[7] Greengard, P. , Phosphorylated proteins as physiological effectors. Science 1978, 199, 146–152. 22932 10.1126/science.22932

[8] Choudhary, C. , Kumar, C. , Gnad, F. , Nielsen, M. L. et al., Lysine acetylation targets protein complexes and co‐regulates major cellular functions. Science 2009, 325, 834–840. 19608861 10.1126/science.1175371

[9] Bouvignies, G. , Vallurupalli, P. , Kay, L. E. , Visualizing side chains of invisible protein conformers by solution NMR. J. Mol. Biol. 2014, 426, 763–774. 24211467 10.1016/j.jmb.2013.10.041

[10] Gavin, A. C. , Aloy, P. , Grandi, P. , Krause, R. et al., Proteome survey reveals modularity of the yeast cell machinery. Nature 2006, 440, 631–636. 16429126 10.1038/nature04532

[11] Hsu, W. L. , Oldfield, C. J. , Xue, B. , Meng, J. et al., Exploring the binding diversity of intrinsically disordered proteins involved in one‐to‐many binding. Protein Sci. 2013, 22, 258–273. 23233352 10.1002/pro.2207 PMC3595456

[12] Bondos, S. E. , Swint‐Kruse, L. , Matthews, K. S. , Flexibility and disorder in gene regulation: LacI/GalR and Hox proteins. J. Biol. Chem. 2015, 290, 24669–24677. 26342073 10.1074/jbc.R115.685032 PMC4598980

[13] Dunker, A. K. , Bondos, S. E. , Huang, F. , Oldfield, C. J. , Intrinsically disordered proteins and multicellular organisms. Semin. Cell Dev. Biol. 2015, 37, 44–55. 25307499 10.1016/j.semcdb.2014.09.025

[14] Toyama, B. H. , Hetzer, M. W. , Protein homeostasis: live long, won't prosper. Nat. Rev. Mol. Cell Biol. 2013, 14, 55–61. 23258296 10.1038/nrm3496 PMC3570024

[15] Eden, E. , Geva‐Zatorsky, N. , Issaeva, I. , Cohen, A. et al., Proteome half‐life dynamics in living human cells. Science 2011, 331, 764–768. 21233346 10.1126/science.1199784

[16] Boisvert, F. M. , Ahmad, Y. , Gierlinski, M. , Charriere, F. et al., A quantitative spatial proteomics analysis of proteome turnover in human cells. Mol. Cell. Proteomics 2012, 11, M111 011429. 10.1074/mcp.M111.011429 PMC3316722 21937730

[17] Gsponer, J. , Futschik, M. E. , Teichmann, S. A. , Babu, M. M. , Tight regulation of unstructured proteins: from transcript synthesis to protein degradation. Science 2008, 322, 1365–1368. 19039133 10.1126/science.1163581 PMC2803065

[18] Dunkley, T. P. , Watson, R. , Griffin, J. L. , Dupree, P. , Lilley, K. S. , Localization of organelle proteins by isotope tagging (LOPIT). Mol. Cell. Proteomics 2004, 3, 1128–1134. 15295017 10.1074/mcp.T400009-MCP200

[19] Smith, L. M. , Kelleher, N. L. , Consortium for top down, P., Proteoform: a single term describing protein complexity. Nat. Methods 2013, 10, 186–187. 23443629 10.1038/nmeth.2369 PMC4114032

[20] Riley, N. M. , Coon, J. J. , Phosphoproteomics in the age of rapid and deep proteome profiling. Anal. Chem. 2016, 88, 74–94. 26539879 10.1021/acs.analchem.5b04123 PMC4790442

[21] Rose, C. M. , Isasa, M. , Ordureau, A. , Prado, M. A. et al., Highly multiplexed quantitative mass spectrometry analysis of ubiquitylomes. Cell Syst. 2016, 3, 395–403 e394. 27667366 10.1016/j.cels.2016.08.009 PMC5241079

[22] Smith, K. T. , Workman, J. L. , Introducing the acetylome. Nat. Biotechnol. 2009, 27, 917–919. 19816449 10.1038/nbt1009-917

[23] Uversky, V. N. , p53 proteoforms and intrinsic disorder: an illustration of the protein structure‐function continuum concept. Int. J. Mol. Sci. 2016, 17, pii: E1874. 10.3390/ijms17111874 PMC5133874 27834926

[24] Dunker, A. K. , Babu, M. M. , Barbar, E. , Blackledge, M. et al., What's in a name? Why these proteins are intrinsically disordered. Intrinsical. Disord. Proteins 2013, 1, e24157. 10.4161/idp.24157 PMC5424803 28516007

[25] Tsvetkov, P. , Asher, G. , Paz, A. , Reuven, N. et al., Operational definition of intrinsically unstructured protein sequences based on susceptibility to the 20S proteasome. Proteins 2008, 70, 1357–1366. 17879262 10.1002/prot.21614

[26] van der Lee, R ., Buljan, M. , Lang, B. , Weatheritt, R. J. et al., Classification of intrinsically disordered regions and proteins. Chem. Rev. 2014, 114, 6589–6631. 24773235 10.1021/cr400525m PMC4095912

[27] Kalodimos, C. G. , Biris, N. , Bonvin, A. M. , Levandoski, M. M. et al., Structure and flexibility adaptation in nonspecific and specific protein‐DNA complexes. Science 2004, 305, 386–389. 15256668 10.1126/science.1097064

[28] Love, J. J. , Li, X. , Chung, J. , Dyson, H. J. , Wright, P. E. , The LEF‐1 high‐mobility group domain undergoes a disorder‐to‐order transition upon formation of a complex with cognate DNA. Biochemistry 2004, 43, 8725–8734. 15236581 10.1021/bi049591m

[29] Theillet, F. X. , Binolfi, A. , Bekei, B. , Martorana, A. et al., Structural disorder of monomeric alpha‐synuclein persists in mammalian cells. Nature 2016, 530, 45–50. 26808899 10.1038/nature16531

[30] Dunker, A. K. , Cortese, M. S. , Romero, P. , Iakoucheva, L. M. , Uversky, V. N. , Flexible nets. The roles of intrinsic disorder in protein interaction networks. FEBS J. 2005, 272, 5129–5148. 16218947 10.1111/j.1742-4658.2005.04948.x

[31] Anvarian, Z. , Nojima, H. , van Kappel, E. C. , Madl, T. et al., Axin cancer mutants form nanoaggregates to rewire the Wnt signaling network. Nat. Struct. Mol. Biol. 2016, 23, 324–332. 26974125 10.1038/nsmb.3191

[32] Minde, D. P. , Radli, M. , Forneris, F. , Maurice, M. M. , Ruediger, S. G. D. , Large extent of disorder in adenomatous polyposis coli offers a strategy to guard Wnt signalling against point mutations. PLoS One 2013, 8, e77257. 24130866 10.1371/journal.pone.0077257 PMC3793970

[33] Minde, D. P. , Anvarian, Z. , Rudiger, S. G. D. , Maurice, M. M. , Messing up disorder: how do missense mutations in the tumor suppressor protein APC lead to cancer? Molecular Cancer 2011, 10, 101. 21859464 10.1186/1476-4598-10-101 PMC3170638

[34] Habchi, J. , Tompa, P. , Longhi, S. , Uversky, V. N. , Introducing protein intrinsic disorder. Chem. Rev. 2014, 114, 6561–6588. 24739139 10.1021/cr400514h

[35] Wright, P. E. , Dyson, H. J. , Intrinsically disordered proteins in cellular signalling and regulation. Nat. Rev. Mol. Cell Biol. 2015, 16, 18–29. 25531225 10.1038/nrm3920 PMC4405151

[36] Lewis, T. E. , Sillitoe, I. , Andreeva, A. , Blundell, T. L. et al., Genome3D: a UK collaborative project to annotate genomic sequences with predicted 3D structures based on SCOP and CATH domains. Nucleic Acids Res. 2013, 41, D499–D507. 23203986 10.1093/nar/gks1266 PMC3531217

[37] Dunker, A. K. , Brown, C. J. , Lawson, J. D. , Iakoucheva, L. M. , Obradovic, Z. , Intrinsic disorder and protein function. Biochemistry 2002, 41, 6573–6582. 12022860 10.1021/bi012159+

[38] Piovesan, D. , Tabaro, F. , Mičetić, I. , Necci, M. et al., DisProt 7.0: a major update of the database of disordered proteins. Nucleic Acids Res. 2016, gkw1056. 10.1093/nar/gkw1056 PMC5210544 27899601

[39] Pande, K. , Hutchison, C. D. , Groenhof, G. , Aquila, A. et al., Femtosecond structural dynamics drives the trans/cis isomerization in photoactive yellow protein. Science 2016, 352, 725–729. 27151871 10.1126/science.aad5081 PMC5291079

[40] Stagno, J. R. , Liu, Y. , Bhandari, Y. R. , Conrad, C. E. et al., Structures of riboswitch RNA reaction states by mix‐and‐inject XFEL serial crystallography. Nature 2016, 541, 242–246. 27841871 10.1038/nature20599 PMC5502819

[41] Burnley, B. T. , Afonine, P. V. , Adams, P. D. , Gros, P. , Modelling dynamics in protein crystal structures by ensemble refinement. Elife 2012, 1, e00311. 23251785 10.7554/eLife.00311 PMC3524795

[42] Richards, M. W. , Burgess, S. G. , Poon, E. , Carstensen, A. et al., Structural basis of N‐Myc binding by Aurora‐A and its destabilization by kinase inhibitors. Proc. Natl. Acad. Sci. U. S. A. 2016, 113, 13726–13731. 27837025 10.1073/pnas.1610626113 PMC5137718

[43] Dauch, D. , Rudalska, R. , Cossa, G. , Nault, J. C. et al., A MYC‐aurora kinase A protein complex represents an actionable drug target in p53‐altered liver cancer. Nat. Med. 2016, 22, 744–753. 27213815 10.1038/nm.4107

[44] Konrat, R. , NMR contributions to structural dynamics studies of intrinsically disordered proteins. J. Magn. Reson. 2014, 241, 74–85. 24656082 10.1016/j.jmr.2013.11.011 PMC3985426

[45] Fischer, N. , Neumann, P. , Konevega, A. L. , Bock, L. V. et al., Structure of the E. coli ribosome‐EF‐Tu complex at <3 A resolution by Cs‐corrected cryo‐EM. Nature 2015, 520, 567–570. 25707802 10.1038/nature14275

[46] Rodriguez, J. A. , Ivanova, M. I. , Sawaya, M. R. , Cascio, D. et al., Structure of the toxic core of alpha‐synuclein from invisible crystals. Nature 2015, 525, 486–490. 26352473 10.1038/nature15368 PMC4791177

[47] Liko, I. , Allison, T. M. , Hopper, J. T. , Robinson, C. V. , Mass spectrometry guided structural biology. Curr. Opin. Struct. Biol. 2016, 40, 136–144. 27721169 10.1016/j.sbi.2016.09.008

[48] Minde, D. P. , Halff, E. F. , Tans, S. , Designing disorder: Tales of the unexpected tails. Intrinsical. Disord. Proteins 2013, 1, 5–4. 10.4161/idp.26790 PMC5424805 28516025

[49] Mittermaier, A. , Kay, L. E. , New tools provide new insights in NMR studies of protein dynamics. Science 2006, 312, 224–228. 16614210 10.1126/science.1124964

[50] Hoelen, H. , Kleizen, B. , Schmidt, A. , Richardson, J. et al., The primary folding defect and rescue of DeltaF508 CFTR emerge during translation of the mutant domain. PLoS One 2010, 5, e15458. 21152102 10.1371/journal.pone.0015458 PMC2994901

[51] Park, C. , Marqusee, S. , Pulse proteolysis: a simple method for quantitative determination of protein stability and ligand binding. Nat. Methods 2005, 2, 207–212. 15782190 10.1038/nmeth740

[52] Kim, M. S. , Song, J. , Park, C. , Determining protein stability in cell lysates by pulse proteolysis and Western blotting. Protein Sci. 2009, 18, 1051–1059. 19388050 10.1002/pro.115 PMC2771307

[53] Schlebach, J. P. , Kim, M. S. , Joh, N. H. , Bowie, J. U. , Park, C. , Probing membrane protein unfolding with pulse proteolysis. J. Mol. Biol. 2011, 406, 545–551. 21192947 10.1016/j.jmb.2010.12.018 PMC3039306

[54] Adhikari, J. , Fitzgerald, M. C. , SILAC‐pulse proteolysis: a mass spectrometry‐based method for discovery and cross‐validation in proteome‐wide studies of ligand binding. J. Am. Soc. Mass Spectrom. 2014, 25, 2073–2083. 25315461 10.1007/s13361-014-0992-y

[55] Minde, D. P. , Maurice, M. M. , Rudiger, S. G. D. , Determining biophysical protein stability in lysates by a fast proteolysis assay, FASTpp. PLoS One 2012, 7, e46147. 23056252 10.1371/journal.pone.0046147 PMC3463568

[56] Robaszkiewicz, K. , Ostrowska, Z. , Cyranka‐Czaja, A. , Moraczewska, J. , Impaired tropomyosin‐troponin interactions reduce activation of the actin thin filament. Biochim. Biophys. Acta. 2015, 1854, 381–390. 25603119 10.1016/j.bbapap.2015.01.004

[57] Miletta, M. C. , Eble, A. , Janner, M. , Parween, S. et al., IGHD II: a novel GH‐1 gene mutation (GH‐L76P) severely affects GH folding, stability, and secretion. J. Clin. Endocrinol. Metab. 2015, 100, E1575–E1583. 26485222 10.1210/jc.2015-3265

[58] Gilbreth, R. N. , Chacko, B. M. , Grinberg, L. , Swers, J. S. , Baca, M. , Stabilization of the third fibronectin type III domain of human tenascin‐C through minimal mutation and rational design. Protein Eng. Des. Sel. 2014, 27, 411–418. 24996411 10.1093/protein/gzu024

[59] Huang, J. R. , Hsu, S. T. , Christodoulou, J. , Jackson, S. E. , The extremely slow‐exchanging core and acid‐denatured state of green fluorescent protein. HFSP J. 2008, 2, 378–387. 19436495 10.2976/1.2976660 PMC2645585

[60] Fontana, A. , de Laureto, P. P. , Spolaore, B. , Frare, E. et al., Probing protein structure by limited proteolysis. Acta. Biochim. Pol. 2004, 51, 299–321. 15218531

[61] Hedstrom, L. , Serine protease mechanism and specificity. Chem. Rev. 2002, 102, 4501–4524. 12475199 10.1021/cr000033x

[62] Feng, Y. , De Franceschi, G. , Kahraman, A. , Soste, M. et al., Global analysis of protein structural changes in complex proteomes. Nat. Biotechnol. 2014, 32, 1036–1044. 25218519 10.1038/nbt.2999

[63] Boersema, P. J. , Kahraman, A. , Picotti, P. , Proteomics beyond large‐scale protein expression analysis. Curr. Opin. Biotechnol. 2015, 34, 162–170. 25636126 10.1016/j.copbio.2015.01.005

[64] Geiger, R. , Rieckmann, J. C. , Wolf, T. , Basso, C. et al., L‐arginine modulates T cell metabolism and enhances survival and anti‐tumor activity. Cell 2016, 167, 829–842 e813. 27745970 10.1016/j.cell.2016.09.031 PMC5075284

[65] Khorasanizadeh, S. , Peters, I. D. , Butt, T. R. , Roder, H. , Folding and stability of a tryptophan‐containing mutant of ubiquitin. Biochemistry 1993, 32, 7054–7063. 8392867 10.1021/bi00078a034

[66] Dusa, A. , Kaylor, J. , Edridge, S. , Bodner, N. et al., Characterization of oligomers during alpha‐synuclein aggregation using intrinsic tryptophan fluorescence. Biochemistry 2006, 45, 2752–2760. 16489768 10.1021/bi051426z

[67] Iacob, R. E. , Engen, J. R. , Hydrogen exchange mass spectrometry: are we out of the quicksand? J. Am. Soc. Mass Spectrom. 2012, 23, 1003–1010. 22476891 10.1007/s13361-012-0377-z PMC3389995

[68] Maaty, W. S. , Weis, D. D. , Label‐free, in‐solution screening of peptide libraries for binding to protein targets using hydrogen exchange mass spectrometry. J. Am. Chem. Soc. 2016, 138, 1335–1343. 26741284 10.1021/jacs.5b11742 PMC5293133

[69] Colin, P. Y. , Kintses, B. , Gielen, F. , Miton, C. M. et al., Ultrahigh‐throughput discovery of promiscuous enzymes by picodroplet functional metagenomics. Nat. Commun. 2015, 6, 10008. 26639611 10.1038/ncomms10008 PMC4686663

[70] Zhang, X. , Less is more: membrane protein digestion beyond urea‐trypsin solution for next‐level proteomics. Mol. Cell. Proteomics 2015, 14, 2441–2453. 26081834 10.1074/mcp.R114.042572 PMC4563727

[71] Pang, Y. , Wang, W. H. , Reid, G. E. , Hunt, D. F. , Bruening, M. L. , Pepsin‐containing membranes for controlled monoclonal antibody digestion prior to mass spectrometry analysis. Anal. Chem. 2015, 87, 10942–10949. 26455365 10.1021/acs.analchem.5b02739 PMC5016144

[72] Mogk, A. , Tomoyasu, T. , Goloubinoff, P. , Rudiger, S. et al., Identification of thermolabile Escherichia coli proteins: prevention and reversion of aggregation by DnaK and ClpB. EMBO J. 1999, 18, 6934–6949. 10601016 10.1093/emboj/18.24.6934 PMC1171757

[73] Mashaghi, A. , Bezrukavnikov, S. , Minde, D. P. , Wentink, A. S. et al., Alternative modes of client binding enable functional plasticity of Hsp70. Nature 2016, 448–451. 10.1038/nature20137 27783598

[74] Jafari, R. , Almqvist, H. , Axelsson, H. , Ignatushchenko, M. et al., The cellular thermal shift assay for evaluating drug target interactions in cells. Nat. Protoc. 2014, 9, 2100–2122. 25101824 10.1038/nprot.2014.138

[75] Savitski, M. M. , Reinhard, F. B. , Franken, H. , Werner, T. et al., Tracking cancer drugs in living cells by thermal profiling of the proteome. Science 2014, 346, 1255784. 25278616 10.1126/science.1255784

[76] Reinhard, F. B. , Eberhard, D. , Werner, T. , Franken, H. et al., Thermal proteome profiling monitors ligand interactions with cellular membrane proteins. Nat. Methods 2015, 12, 1129–1131. 26524241 10.1038/nmeth.3652

[77] Hein, M. Y. , Hubner, N. C. , Poser, I. , Cox, J. et al., A human interactome in three quantitative dimensions organized by stoichiometries and abundances. Cell 2015, 163, 712–723. 26496610 10.1016/j.cell.2015.09.053

[78] Chivers, C. E. , Crozat, E. , Chu, C. , Moy, V. T. et al., A streptavidin variant with slower biotin dissociation and increased mechanostability. Nat. Methods 2010, 7, 391–393. 20383133 10.1038/nmeth.1450 PMC2862113

[79] Demarest, S. J. , Martinez‐Yamout, M. , Chung, J. , Chen, H. et al., Mutual synergistic folding in recruitment of CBP/p300 by p160 nuclear receptor coactivators. Nature 2002, 415, 549–553. 11823864 10.1038/415549a

[80] Chakravarti, D. , LaMorte, V. J. , Nelson, M. C. , Nakajima, T. et al., Role of CBP/P300 in nuclear receptor signalling. Nature 1996, 383, 99–103. 8779723 10.1038/383099a0

[81] Dyson, H. J. , Wright, P. E. , Role of intrinsic protein disorder in the function and interactions of the transcriptional coactivators CREB‐binding protein (CBP) and p300. J. Biol. Chem. 2016, 291, 6714–6722. 26851278 10.1074/jbc.R115.692020 PMC4807259

[82] Smits, A. H. , Vermeulen, M. , Characterizing protein‐protein interactions using mass spectrometry: challenges and opportunities. Trends Biotechnol. 2016, 34, 825–834. 26996615 10.1016/j.tibtech.2016.02.014

[83] Rees, J. S. , Lowe, N. , Armean, I. M. , Roote, J. et al., In vivo analysis of proteomes and interactomes using Parallel Affinity Capture (iPAC) coupled to mass spectrometry. Mol. Cell. Proteomics 2011, 10, M110 002386. 10.1074/mcp.M110.002386 PMC3108830 21447707

[84] Rees, J. S. , Lilley, K. S. , Jackson, A. P. , SILAC‐iPAC: a quantitative method for distinguishing genuine from non‐specific components of protein complexes by parallel affinity capture. J. Proteomics 2015, 115, 143–156. 25534881 10.1016/j.jprot.2014.12.006 PMC4329988

[85] Hubner, N. C. , Bird, A. W. , Cox, J. , Splettstoesser, B. et al., Quantitative proteomics combined with BAC TransgeneOmics reveals in vivo protein interactions. J. Cell Biol. 2010, 189, 739–754. 20479470 10.1083/jcb.200911091 PMC2872919

[86] Gu, L. , Li, C. , Aach, J. , Hill, D. E. et al., Multiplex single‐molecule interaction profiling of DNA‐barcoded proteins. Nature 2014, 515, 554–557. 25252978 10.1038/nature13761 PMC4246050

[87] Wachsmuth, M. , Conrad, C. , Bulkescher, J. , Koch, B. et al., High‐throughput fluorescence correlation spectroscopy enables analysis of proteome dynamics in living cells. Nat. Biotechnol. 2015, 33, 384–389. 25774713 10.1038/nbt.3146

[88] Kao, A. , Chiu, C. L. , Vellucci, D. , Yang, Y. et al., Development of a novel cross‐linking strategy for fast and accurate identification of cross‐linked peptides of protein complexes. Mol. Cell. Proteomics 2011, 10, M110 002212. 10.1074/mcp.M110.002212 PMC3013449 20736410

[89] Lossl, P. , van de Waterbeemd, M. , Heck, A. J. , The diverse and expanding role of mass spectrometry in structural and molecular biology. EMBO J. 2016. 10.15252/embj.201694818 PMC5167345 27797822

[90] Leitner, A. , Faini, M. , Stengel, F. , Aebersold, R. , Crosslinking and Mass Spectrometry: An integrated technology to understand the structure and function of molecular machines. Trends Biochem. Sci. 2016, 41, 20–32. 26654279 10.1016/j.tibs.2015.10.008

[91] Liu, F. , Rijkers, D. T. , Post, H. , Heck, A. J. , Proteome‐wide profiling of protein assemblies by cross‐linking mass spectrometry. Nat. Methods 2015, 12, 1179–1184. 26414014 10.1038/nmeth.3603

[92] Herzog, F. , Kahraman, A. , Boehringer, D. , Mak, R. et al., Structural probing of a protein phosphatase 2A network by chemical cross‐linking and mass spectrometry. Science 2012, 337, 1348–1352. 22984071 10.1126/science.1221483

[93] Kaake, R. M. , Wang, X. , Burke, A. , Yu, C. et al., A new in vivo cross‐linking mass spectrometry platform to define protein‐protein interactions in living cells. Mol. Cell. Proteomics 2014, 13, 3533–3543. 25253489 10.1074/mcp.M114.042630 PMC4256503

[94] Navare, A. T. , Chavez, J. D. , Zheng, C. , Weisbrod, C. R. et al., Probing the protein interaction network of Pseudomonas aeruginosa cells by chemical cross‐linking mass spectrometry. Structure 2015, 23, 762–773. 25800553 10.1016/j.str.2015.01.022 PMC4756656

[95] Hutchison, C. A., 3rd , Chuang, R. Y. , Noskov, V. N. , Assad‐Garcia, N. et al., Design and synthesis of a minimal bacterial genome. Science 2016, 351, aad6253. 10.1126/science.aad6253 27013737

[96] Papanastasiou, M. , Orfanoudaki, G. , Koukaki, M. , Kountourakis, N. et al., The Escherichia coli peripheral inner membrane proteome. Mol. Cell. Proteomics 2013, 12, 599–610. 23230279 10.1074/mcp.M112.024711 PMC3591654

[97] Wu, M. M. , Llopis, J. , Adams, S. , McCaffery, J. M. et al., Organelle pH studies using targeted avidin and fluorescein‐biotin. Chem. Biol. 2000, 7, 197–209. 10712929 10.1016/s1074-5521(00)00088-0

[98] Blamowska, M. , Neupert, W. , Hell, K. , Biogenesis of the mitochondrial Hsp70 chaperone. J. Cell Biol. 2012, 199, 125–135. 23007651 10.1083/jcb.201205012 PMC3461517

[99] Lam, S. S. , Martell, J. D. , Kamer, K. J. , Deerinck, T. J. et al., Directed evolution of APEX2 for electron microscopy and proximity labeling. Nat. Methods 2015, 12, 51–54. 25419960 10.1038/nmeth.3179 PMC4296904

[100] Loh, K. H. , Stawski, P. S. , Draycott, A. S. , Udeshi, N. D. et al., Proteomic analysis of unbounded cellular compartments: synaptic clefts. Cell 2016, 166, 1295–1307 e1221. 27565350 10.1016/j.cell.2016.07.041 PMC5167540

[101] Rees, J. S. , Li, X. W. , Perrett, S. , Lilley, K. S. , Jackson, A. P. , Selective proteomic proximity labeling assay using tyramide (SPPLAT): a quantitative method for the proteomic analysis of localized membrane‐bound protein clusters. Curr. Protoc. Protein Sci. 2015, 80, 19 27 11–18. 10.1002/0471140864.ps1927s80 25829300

[102] Li, X. W. , Rees, J. S. , Xue, P. , Zhang, H. et al., New insights into the DT40 B cell receptor cluster using a proteomic proximity labeling assay. J. Biol. Chem. 2014, 289, 14434–14447. 24706754 10.1074/jbc.M113.529578 PMC4031500

[103] Rees, J. S. , Li, X. W. , Perrett, S. , Lilley, K. S. , Jackson, A. P. , Protein neighbors and proximity proteomics. Mol. Cell. Proteomics 2015, 14, 2848–2856. 26355100 10.1074/mcp.R115.052902 PMC4638030

[104] Chapman‐Smith, A. , Cronan, J. E., Jr. , The enzymatic biotinylation of proteins: a post‐translational modification of exceptional specificity. Trends Biochem. Sci. 1999, 24, 359–363. 10470036 10.1016/s0968-0004(99)01438-3

[105] Roux, K. J. , Kim, D. I. , Burke, B. , BioID: a screen for protein‐protein interactions. Curr. Protoc. Protein Sci. 2013, 74, Unit 19 23. 10.1002/0471140864.ps1923s74 24510646

[106] Tron, C. M. , McNae, I. W. , Nutley, M. , Clarke, D. J. et al., Structural and functional studies of the biotin protein ligase from Aquifex aeolicus reveal a critical role for a conserved residue in target specificity. J. Mol. Biol. 2009, 387, 129–146. 19385043 10.1016/j.jmb.2008.12.086

[107] Kim, D. I. , Jensen, S. C. , Noble, K. A. , Kc, B. et al., An improved smaller biotin ligase for BioID proximity labeling. Mol. Biol. Cell 2016, 27, 1188–1196. 26912792 10.1091/mbc.E15-12-0844 PMC4831873

[108] Schiapparelli, L. M. , McClatchy, D. B. , Liu, H. H. , Sharma, P. et al., Direct detection of biotinylated proteins by mass spectrometry. J. Proteome Res. 2014, 13, 3966–3978. 25117199 10.1021/pr5002862 PMC4156236

[109] Reck‐Peterson, S. L. , Yildiz, A. , Carter, A. P. , Gennerich, A. et al., Single‐molecule analysis of dynein processivity and stepping behavior. Cell 2006, 126, 335–348. 16873064 10.1016/j.cell.2006.05.046 PMC2851639

[110] Boke, E. , Ruer, M. , Wuhr, M. , Coughlin, M. et al., Amyloid‐like self‐assembly of a cellular compartment. Cell 2016, 166, 637–650. 27471966 10.1016/j.cell.2016.06.051 PMC5082712

[111] Brangwynne, C. P. , Tompa, P. , Pappu, R. V. , Polymer physics of intracellular phase transitions. Nat. Phys. 2015, 11, 899–904.

[112] Oh, E. , Becker, A. H. , Sandikci, A. , Huber, D. et al., Selective ribosome profiling reveals the cotranslational chaperone action of trigger factor in vivo. Cell 2011, 147, 1295–1308. 22153074 10.1016/j.cell.2011.10.044 PMC3277850

[113] Andersen, J. S. , Wilkinson, C. J. , Mayor, T. , Mortensen, P. et al., Proteomic characterization of the human centrosome by protein correlation profiling. Nature 2003, 426, 570–574. 14654843 10.1038/nature02166

[114] Christoforou, A. , Mulvey, C. M. , Breckels, L. M. , Geladaki, A. et al., A draft map of the mouse pluripotent stem cell spatial proteome. Nat. Commun. 2016, 7, 8992. 26754106 10.1038/ncomms9992 PMC4729960

[115] Itzhak, D. N. , Tyanova, S. , Cox, J. , Borner, G. H. , Global, quantitative and dynamic mapping of protein subcellular localization. Elife 2016, 5, pii: e16950. 10.7554/eLife.16950 PMC4959882 27278775

[116] Preitner, N. , Quan, J. , Nowakowski, D. W. , Hancock, M. L. et al., APC is an RNA‐binding protein, and its interactome provides a link to neural development and microtubule assembly. Cell 2014, 158, 368–382. 25036633 10.1016/j.cell.2014.05.042 PMC4133101

[117] Li, V. S. , Ng, S. S. , Boersema, P. J. , Low, T. Y. et al., Wnt signaling through inhibition of beta‐catenin degradation in an intact Axin1 complex. Cell 2012, 149, 1245–1256. 22682247 10.1016/j.cell.2012.05.002

[118] Gatto, L. , Breckels, L. M. , Wieczorek, S. , Burger, T. , Lilley, K. S. , Mass‐spectrometry‐based spatial proteomics data analysis using pRoloc and pRolocdata. Bioinformatics 2014, 30, 1322–1324. 24413670 10.1093/bioinformatics/btu013 PMC3998135

[119] Medalia, O. , Weber, I. , Frangakis, A. S. , Nicastro, D. et al., Macromolecular architecture in eukaryotic cells visualized by cryoelectron tomography. Science 2002, 298, 1209–1213. 12424373 10.1126/science.1076184

[120] Fernandez‐Busnadiego, R. , Zuber, B. , Maurer, U. E. , Cyrklaff, M. et al., Quantitative analysis of the native presynaptic cytomatrix by cryoelectron tomography. J. Cell Biol. 2010, 188, 145–156. 20065095 10.1083/jcb.200908082 PMC2812849

[121] Beck, M. , Malmstrom, J. A. , Lange, V. , Schmidt, A. et al., Visual proteomics of the human pathogen Leptospira interrogans. Nat Meth. 2009, 6, 817–823. 10.1038/nmeth.1390 PMC2862215 19838170

[122] Nixon‐Abell, J. , Obara, C. J. , Weigel, A. V. , Li, D. et al., Increased spatiotemporal resolution reveals highly dynamic dense tubular matrices in the peripheral ER. Science 2016, 354, pii: aaf3928. 10.1126/science.aaf3928 PMC6528812 27789813

[123] Ku, T. , Swaney, J. , Park, J. Y. , Albanese, A. et al., Multiplexed and scalable super‐resolution imaging of three‐dimensional protein localization in size‐adjustable tissues. Nat. Biotechnol. 2016, 34, 973–981. 27454740 10.1038/nbt.3641 PMC5070610

[124] Crick, F. , Diffusion in embryogenesis. Nature 1970, 225, 420–422. 5411117 10.1038/225420a0

[125] Farin, H. F. , Jordens, I. , Mosa, M. H. , Basak, O. et al., Visualization of a short‐range Wnt gradient in the intestinal stem‐cell niche. Nature 2016, 530, 340–343. 26863187 10.1038/nature16937

[126] Xue, B. , Dunker, A. K. , Uversky, V. N. , The roles of intrinsic disorder in orchestrating the Wnt‐pathway. J. Biomol. Struct. Dyn. 2012, 29, 843–861. 22292947 10.1080/073911012010525024

[127] Rosenberg, M. M. , Yang, F. , Mohn, J. L. , Storer, E. K. , Jacob, M. H. , The postsynaptic adenomatous polyposis coli (APC) multiprotein complex is required for localizing neuroligin and neurexin to neuronal nicotinic synapses in vivo. J. Neurosci. 2010, 30, 11073–11085. 20720115 10.1523/JNEUROSCI.0983-10.2010 PMC2945243

[128] Heijs, B. , Carreira, R. J. , Tolner, E. A. , de Ru, A. H. et al., Comprehensive analysis of the mouse brain proteome sampled in mass spectrometry imaging. Anal. Chem. 2015, 87, 1867–1875. 25535922 10.1021/ac503952q

[129] Hopf, T. A. , Colwell, L. J. , Sheridan, R. , Rost, B. et al., Three‐dimensional structures of membrane proteins from genomic sequencing. Cell 2012, 149, 1607–1621. 22579045 10.1016/j.cell.2012.04.012 PMC3641781

[130] Ovchinnikov, S. , Kamisetty, H. , Baker, D. , Robust and accurate prediction of residue‐residue interactions across protein interfaces using evolutionary information. Elife 2014, 3, e02030. 24842992 10.7554/eLife.02030 PMC4034769

[131] Toth‐Petroczy, A. , Palmedo, P. , Ingraham, J. , Hopf, T. A. et al., Structured states of disordered proteins from genomic sequences. Cell 2016, 167, 158–170 e112. 27662088 10.1016/j.cell.2016.09.010 PMC5451116

[132] Karplus, M. , McCammon, J. A. , Molecular dynamics simulations of biomolecules. Nat. Struct. Biol. 2002, 9, 646–652. 12198485 10.1038/nsb0902-646

[133] Moult, J. , Fidelis, K. , Kryshtafovych, A. , Schwede, T. , Tramontano, A. , Critical assessment of methods of protein structure prediction: progress and new directions in round XI. Proteins 2016, 84 Suppl 1, 4–14. 27171127 10.1002/prot.25064 PMC5394799

[134] Huang, J. , Rauscher, S. , Nawrocki, G. , Ran, T. et al., CHARMM36m: an improved force field for folded and intrinsically disordered proteins. Nat. Methods 2016, 71–73. 27819658 10.1038/nmeth.4067 PMC5199616

[135] Rauscher, S. , Gapsys, V. , Gajda, M. J. , Zweckstetter, M. et al., Structural ensembles of intrinsically disordered proteins depend strongly on force field: a comparison to experiment. J. Chem. Theory Comput. 2015, 11, 5513–5524. 26574339 10.1021/acs.jctc.5b00736

[136] Breckels, L. M. , Holden, S. B. , Wojnar, D. , Mulvey, C. M. et al., Learning from heterogeneous data sources: an application in spatial proteomics. PLoS Comput. Biol. 2016, 12, e1004920. 27175778 10.1371/journal.pcbi.1004920 PMC4866734

[137] Ebrahim, A. , Brunk, E. , Tan, J. , O'Brien, E. J. et al., Multi‐omic data integration enables discovery of hidden biological regularities. Nat Commun. 2016, 7, 13091. 27782110 10.1038/ncomms13091 PMC5095171

[138] Eddy, S. R. , The C‐value paradox, junk DNA and ENCODE. Curr. Biol. 2012, 22, R898–899. 23137679 10.1016/j.cub.2012.10.002

[139] Doolittle, W. F. , Is junk DNA bunk? A critique of ENCODE. Proc. Natl. Acad. Sci. U. S. A. 2013, 110, 5294–5300. 23479647 10.1073/pnas.1221376110 PMC3619371

[140] Elowitz, M. B. , Levine, A. J. , Siggia, E. D. , Swain, P. S. , Stochastic gene expression in a single cell. Science 2002, 297, 1183–1186. 12183631 10.1126/science.1070919

[141] Kiviet, D. J. , Nghe, P. , Walker, N. , Boulineau, S. et al., Stochasticity of metabolism and growth at the single‐cell level. Nature 2014, 514, 376–379. 25186725 10.1038/nature13582

[142] Schwander, T. , Schada von Borzyskowski, L. , Burgener, S. , Cortina, N. S. , Erb, T. J. , A synthetic pathway for the fixation of carbon dioxide in vitro. Science 2016, 354, 900–904. 27856910 10.1126/science.aah5237 PMC5892708

[143] Kromdijk, J. , Glowacka, K. , Leonelli, L. , Gabilly, S. T. et al., Improving photosynthesis and crop productivity by accelerating recovery from photoprotection. Science 2016, 354, 857–861. 27856901 10.1126/science.aai8878

[144] Hebert, A. S. , Richards, A. L. , Bailey, D. J. , Ulbrich, A. et al., The one hour yeast proteome. Mol. Cell. Proteomics 2014, 13, 339–347. 24143002 10.1074/mcp.M113.034769 PMC3879625

